# Isolated pulmonary metastatic disease from prostate cancer as an initial symptom in a patient following transurethral resection of the prostate for benign disease with normal histopathology

**DOI:** 10.1093/jscr/rjaf235

**Published:** 2025-04-24

**Authors:** Georgios Tsakaldimis, Dimitrios Diamantidis, George Pappas-Gogos, Pipitsa Valsamaki, Francesk Mulita, Stilianos Giannakopoulos, Christos Kalaitzis

**Affiliations:** Department of Urology, Democritus University of Thrace, Dragana, 68100 Alexandroupolis, Greece; Department of Urology, Democritus University of Thrace, Dragana, 68100 Alexandroupolis, Greece; Department of Surgery, University General Hospital of Alexandroupolis, Dragana, 68100 Alexandroupolis, Greece; Nuclear Medicine Department, Medical School, Democritus University of Thrace, Dragana, 68100 Alexandroupolis, Greece; Department of Surgery, General University Hospital, Rio, 26504 Patras, Greece; Department of Urology, Democritus University of Thrace, Dragana, 68100 Alexandroupolis, Greece; Department of Urology, Democritus University of Thrace, Dragana, 68100 Alexandroupolis, Greece

**Keywords:** prostate cancer, transurethral resection, pulmonary metastases, incidental prostate cancer

## Abstract

Benign prostatic hyperplasia (BPH) remains a predominant cause of acute urinary retention (AUR) in elderly males. Transurethral resection of the prostate (TURP) is the gold-standard treatment for BPH, often uncovering incidental prostate cancer (IPC). Here, we present a unique case of an 80-year-old patient with refractory AUR due to BPH who underwent TURP. Despite benign histopathology, the patient developed isolated pulmonary metastases as the initial symptom of prostate cancer (PCa) eight months postoperatively. Diagnostic evaluation revealed advanced PCa with a Gleason Score of 8 and no other metastases. The rapid progression suggests potential vascular dissemination during TURP, although evidence remains inconclusive. This case highlights the importance of diligent follow-up post-TURP to exclude occult PCa, particularly in high-risk patients. The findings also raise questions about current histopathological evaluation protocols and the need for standardized follow-up practices to improve early detection and management of advanced PCa.

## Introduction

Benign prostatic hyperplasia (BPH) is the most common cause of acute urinary retention (AUR) due to obstruction, at over 53%. Appropriate management includes urinary drainage with a bladder catheter, treatment with α-blockers, and trial without catheter (TWOC) after 3–5 days [1]. Failure of treatment with refractory urinary retention is a classic indication for surgical intervention in patients with BPH. Excluding prostate cancer (PCa) in patients with BPH and an indwelling bladder catheter based on medical history, digital rectal examination of the prostate (DRE), and PSA measurement. However, we cannot rule out the coexistence of PCa with accuracy, as 5%–11% of patients are diagnosed with incidental prostate cancer (IPC) following surgical treatment for BPH [2]. To date, transurethral prostatectomy (TURP) is the gold standard minimally invasive procedure for the treatment of BPH patients. Although TURP does not adversely affect patients with IPC, and many of them, especially old patients, may be managed with active surveillance (AS), the oncological outcomes in those with advanced PCa are controversial in the literature [3–6]. The impact of TURP in patients with inapparent occult PCa is still unknown.

We present the case of a patient with BPH who underwent TURP for refractory urinary retention. Despite a benign pathology report, eight months later, he presented with multiple pulmonary metastases as the initial symptom of PCa.

## Case report

An 80-year-old patient with a history of BPH and a previous negative biopsy 8 years ago due to the elevated value of PSA (PSA = 9.6 ng/ml) presented to our hospital’s emergency department with AUR. During the evaluation, the patient had a large prostate volume ≥ 75 cc on ultrasound, without a suspicious DRE test, with latest PSA value of 3.5 ng/ml a year ago, and was in treatment with a combination of dutasteride/tamsulosin. After two unsuccessful TWOC, the patient underwent TURP. His postoperative course was uneventful, and the patient was discharged from our clinic on the second postoperative day without a bladder catheter. The histological report demonstrates a 50 cc volume surgical specimen with adenomatous hyperplasia of the prostate.

**Figure 1:**
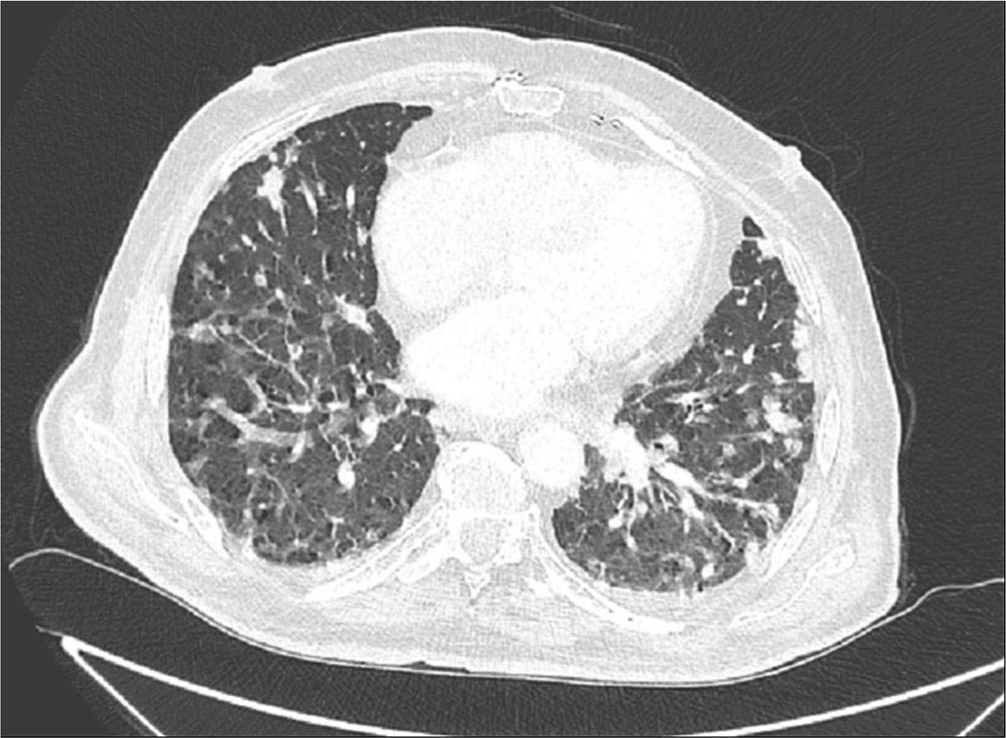
Chest CT revealed multiple pulmonary nodules.

**Figure 2:**
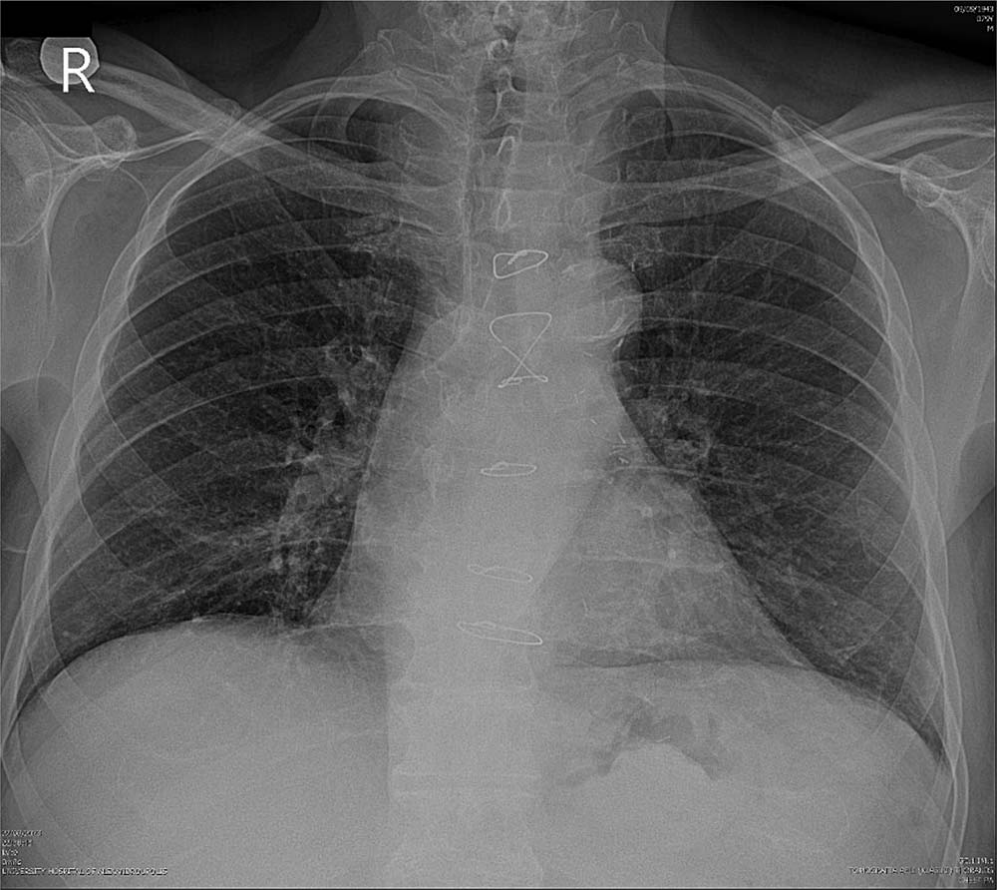
Preoperative chest X-ray.

**Figure 3:**
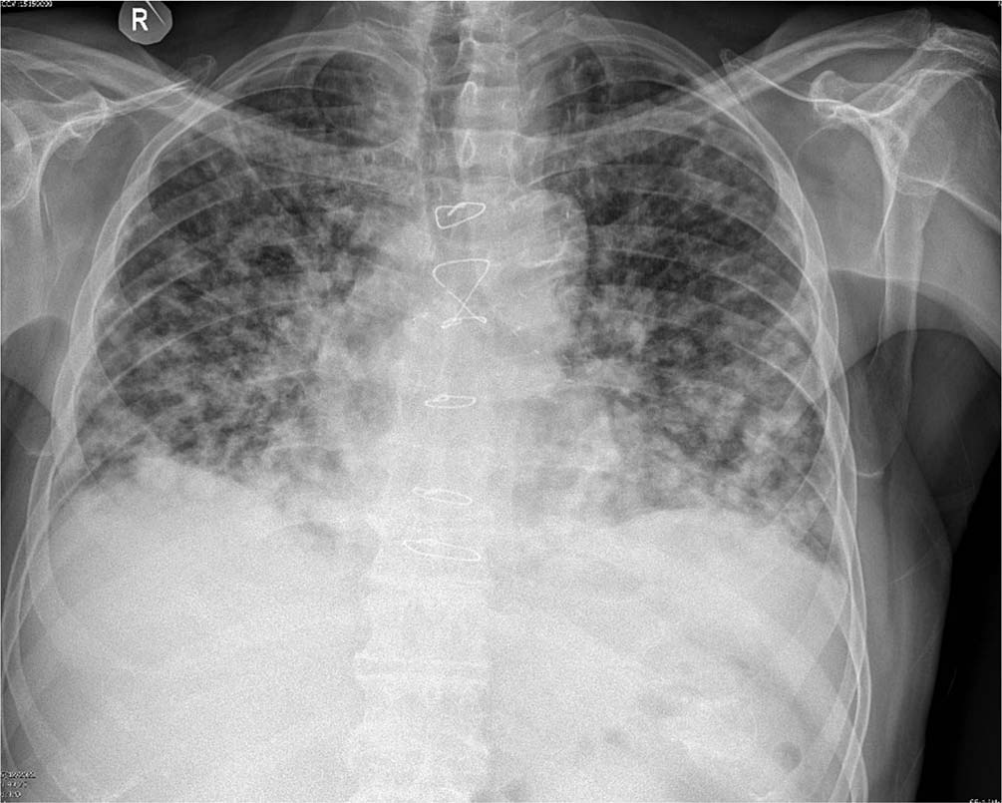
Chest X-ray eight months after TURP.

**Figure 4:**
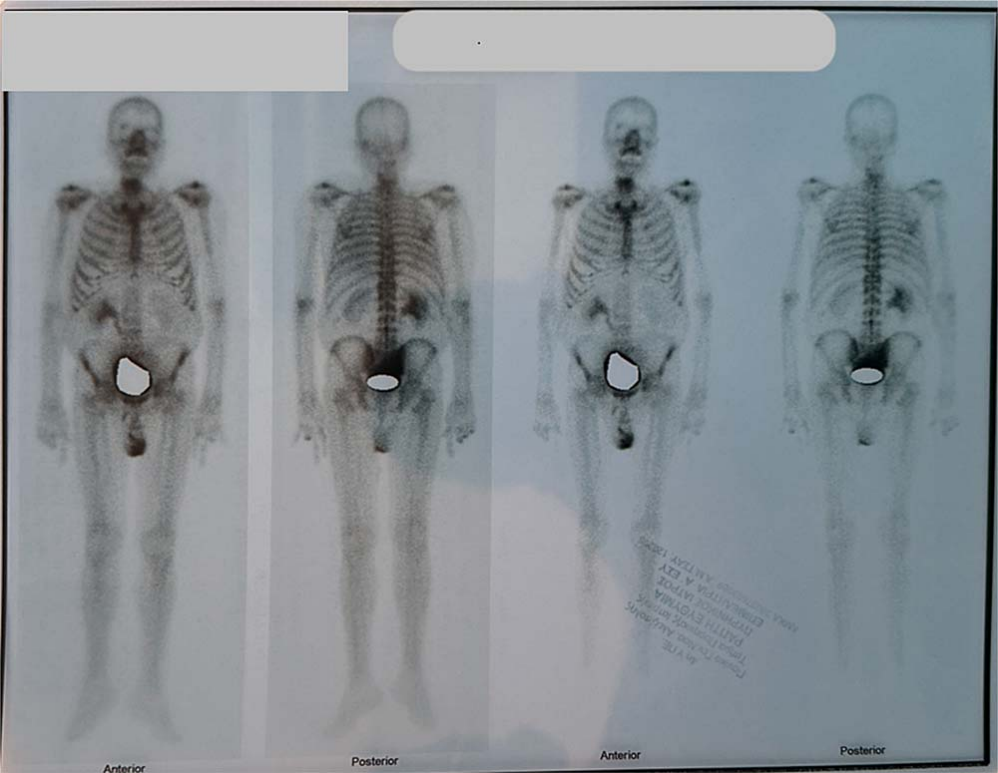
Bone scan was negative for metastatic lesions in bones.

Due to improvement in his symptoms, the patient did not attend his three-month follow-up appointment. Eight months later, the patient was hospitalized with shortness of breath and multiple pulmonary nodules (Fig. 1) on the chest computed tomography (CT) in the pulmonology department of our hospital. Figures 2 and 3 show the preoperative chest X-ray and the chest X-ray taken eight months after the TURP, respectively. Suspected of metastatic disease, a urological evaluation was performed, which revealed an abnormal DRE and a significantly elevated PSA of 134 ng/ml. Diagnostic bronchoscopy and histological findings of the lung biopsy confirmed prostate adenocarcinoma (PCa). TRUS-Bx prostate biopsy (12 cores) confirmed the diagnosis of PCa with involvement of all prostatic cores and Gleason Score (GS) = 8 (4 + 4). Bone scan (Fig. 4) and CT scan were negative for other secondary metastatic lesions in bones and lymph nodes. The patient immediately started combination therapy ADT (degarelix) with apalutamide, and 3 months later, he was off oxygen, and his PSA level had decreased to 14.9 ng/ml.

## Discussion

Excluding the coexistence of PCa in elderly patients with persistent urinary retention and BPH is often a challenge for clinicians, which probably will not change the initial management of patients regarding voiding restoration. In such cases, the patient typically has an indwelling bladder catheter, and the diagnosis of BPH is based on medical history, assessment of prostate volume (Pvol), and DRE. Regarding PSA testing, international guidelines do not recommend routine testing in asymptomatic patients over 76 years old or symptomatic patients with LUTS when the results are unlikely to change the patient’s management [7].

Finding IPC in histological specimen after surgery for BPH is not uncommon, and the management of these patients depends on age, Gleason score, postoperative value of PSA, and clinical assessment at the follow-up [2, 8]. Evaluation of PSA value and DRE after 3 months in the follow-up would have probably detected the disease in our patient, but he omitted the appointment.

TURP is a standard-of-care surgical procedure for symptomatic patients with urinary obstruction due to BPH who do not respond to medical therapy. Standard practice following TURP for BPH is histopathological evaluation of resected specimens, but there is still no consensus on the amount of tissue to be evaluated under the microscope. Evaluating the entire surgical specimen increases IPC detection rates but also raises costs and laboratory workload [9, 10]. Everyday practice is random sampling evaluation of surgical specimens, which allows the detection of over 90% of cases with IPC. The College of American Pathologists recommends testing the entire material for a specimen weighing <12 g and one cassette for each additional 5 g of tissue when the surgical specimen weighs >12 g. In our case, the total volume of the resection specimen was 50 cc, and microscopic examination was performed on a random sample of submitted tissue (random chips) [11].

Previous studies showed that TURP is unlikely to miss clinically significant PCa [12]. A similar result was reported in a recent population-based analysis by Hilscher *et al.*, in which they concluded that the risk of clinically significant PCa after TURP with normal histology is minimal and comparable to that of biopsy [8]. We assume that the failure to detect the tumor in the patient’s histopathology specimen was due either to an incorrect evaluation technique or to the absence of prostatic adenocarcinoma in the submitted sample because the cancer has been in the peripheral zone (PZ) of the prostate which is the most common site of PCa.

The rapid spread of the disease to the lungs, particularly as isolated metastatic disease, may be associated with TURP. Several studies suggest that TURP may promote the vascular spread of cancer cells, leading to distant metastases [13, 14]. The effect of TURP on PCa patients in the literature remains controversial. Several reports in the literature support that TURP is safe and can be applied palliatively in PCa patients in combination with adjuvant therapy [4, 5]. However, long-term oncological data from the population-matched study showed that TURP in patients with mPCa relieves the symptoms of obstruction but reduces the patients’ overall survival (OS) and cancer-specific survival (CSS) [6].

To our knowledge, this is the first report of a patient with isolated lung metastatic disease from undiagnosed PCa following TURP for benign reasons and normal histology.

## Conclusion

It is not always possible to exclude the coexistence of PCa in patients with persistent urinary retention who are led to transurethral resection for benign disease. Follow-up of patients after TURP is essential, as it is a part of the treatment and should not be omitted, regardless of patient age.

## References

[1] Sandhu JS, Bixler BR, Dahm P, et al. Management of lower urinary tract symptoms attributed to benign prostatic hyperplasia (BPH): AUA guideline amendment 2023. J Urol 2024;211:11–9. 10.1097/JU.0000000000003698. 37706750

[2] Capogrosso P, Capitanio U, Vertosick EA, et al. Temporal trend in incidental prostate cancer detection at surgery for benign prostatic hyperplasia. Urology 2018;122:152–7. 10.1016/j.urology.2018.07.028. 30138683 PMC6724539

[3] Hagmann S, Ramakrishnan V, Tamalunas A, et al. Two decades of active surveillance for prostate cancer in a single-Center cohort: favorable outcomes after transurethral resection of the prostate. Cancers (Basel) 2022;14:368. 10.3390/cancers14020368. 35053530 PMC8773913

[4] Qu M, Zhu F, Chen H, et al. Palliative transurethral resection of the prostate in patients with metastatic prostate cancer: a prospective study of 188 patients. J Endourol 2019;33:570–5. 10.1089/end.2019.0108. 31025578

[5] Zhang X, Xia Q, Xu J. Palliative TURP combined with intermittent ADT is a curative therapy to some elderly men with localized prostate adenocarcinoma. J Cancer 2023;14:1232–41. 10.7150/jca.83825. 37215449 PMC10197938

[6] Fang K, Song P, Zhang J, et al. The impact of palliative transurethral resection of the prostate on the prognosis of patients with bladder outlet obstruction and metastatic prostate cancer: a population-matched study. Front Surg 2021;8:726534. 10.3389/fsurg.2021.726534. 34778357 PMC8586220

[7] Baboudjian M, Hashim H, Bhatt N, et al. Summary paper on underactive bladder from the European Association of Urology guidelines on non-neurogenic male lower urinary tract symptoms. Eur Urol 2024;86:213–20. 10.1016/j.eururo.2024.04.004. 38644139

[8] Hilscher M, Røder A, Helgstrand JT, et al. Risk of prostate cancer and death after benign transurethral resection of the prostate-a 20-year population-based analysis. Cancer 2022;128:3674–80. 10.1002/cncr.34407. 35975979 PMC9804454

[9] Newman AJ, Graham MA, Carlton CE, et al. Incidental carcinoma of the prostate at the time of transurethral resection: importance of evaluating every chip. J Urol 1982;128:948–50. 10.1016/S0022-5347(17)53293-0. 6184491

[10] Perera M, Lawrentschuk N, Perera N, et al. Incidental prostate cancer in transurethral resection of prostate specimens in men aged up to 65 years. Prostate Int 2016;4:11–4. 10.1016/j.prnil.2015.10.016. 27014658 PMC4789333

[11] Paner GP, Srigley JR, Harik LR, et al. With guidance from the CAP Cancer and CAP Pathology Electronic Reporting Committees. In: Protocol for the Examination of TURP and Enucleation Specimens from Patients with Carcinoma of the Prostate Gland. Version: 4.2.0.0. 2023,

[12] Hammarsten J, Andersson S, Peeker R, et al. Does transurethral resection of a clinically benign prostate gland increase the risk of developing clinical prostate cancer? A 10-year follow-up study. Cancer 1994;74:2347–51. 10.1002/1097-0142(19941015)74:8<2347::AID-CNCR2820740820>3.0.CO;2-6. 7522950

[13] Heung YM, Walsh K, Sriprasad S, et al. The detection of prostate cells by the reverse transcription-polymerase chain reaction in the circulation of patients undergoing transurethral resection of the prostate. BJU Int 2000;85:65–9. 10.1046/j.1464-410x.2000.00380.x. 10619948

[14] Eschwège P, Moutereau S, Droupy S, et al. Prognostic value of prostate circulating cells detection in prostate cancer patients: a prospective study. Br J Cancer 2009;100:608–10. 10.1038/sj.bjc.6604912. 19223910 PMC2653745

